# Humor in library instruction: a narrative review with implications for the health sciences

**DOI:** 10.5195/jmla.2019.608

**Published:** 2019-07-01

**Authors:** Elena Azadbakht

**Affiliations:** Health Sciences Librarian and Assistant Professor, Mathewson-IGT Knowledge Center, University of Nevada, Reno, NV, eazadbakht@unr.edu

## Abstract

**Objective:**

The review sought to gain a better understanding of humor’s use and impact as a teaching and learning strategy in academic library and health sciences instruction and to determine if the most common techniques across both disciplines can be adapted to increase engagement in medical libraries’ information literacy efforts.

**Methods:**

This narrative review involved retrieving citations from several subject databases, including Library, Information Science & Technology Abstracts; Information Science & Technology Abstracts; Library & Information Science Source; PubMed; and CINAHL. The author limited her review to those publications that explicitly addressed the use of humor in relation to some form of academic library or health sciences instruction. Studies examining use of humor in patient education were excluded.

**Results:**

Scholars and practitioners have consistently written about humor as an instructional strategy from the 1980s onward, in both the library literature and health sciences literature. These authors have focused on instructors’ attitudes, benefits to students, anecdotes, and best practices summaries. Overall, both librarians and health sciences educators have a positive opinion of humor, and many instructors make use of it in their classrooms, though caution and careful planning is advised.

**Conclusions:**

Commonalities between the library and information science literature and health sciences literature provide a cohesive set of best practices and strategies for successfully incorporating comedy into library instruction sessions. Health sciences librarians can adapt several of the most commonly used types of instructional humor (e.g., silly examples, cartoons, storytelling, etc.) to their own contexts with minimal risk.

## INTRODUCTION

The best teachers employ various strategies to keep their students animated and engaged. One such strategy is the use of humor. Although they often visit a particular class or group of students only once, many academic health sciences librarians teach and, therefore, need ways of keeping their audiences interested, especially given the limited amount of time librarians have to build rapport with any one group of students. The topic has captured librarians’ interest since the mid-1980s and continues to be important to the field [1, 2], as evidenced by Perret’s 2016 survey of librarians’ use of humor [4] and Vossler and Sheidlower’s book on humor in library instruction [4]. Humor in teaching has followed a similar trajectory in the health sciences, with a chapter of Robinson’s 1990 book serving as an early example [5].

As of yet, however, no one has compared librarians’ use of humor with that of health sciences instructors. In this review, the library and information science literature and health sciences education literature were examined for discussions and examples of the use of humor in instruction. The commonalities between the two bodies of literature point to widespread beliefs regarding humor’s impact on learning. They also illuminate methods for developing a sense of humor and judiciously using comedy to engage students during the instructional scenarios in which librarians routinely find themselves.

## METHODS

A search of the library and information science literature was conducted in August 2016 and repeated in June 2018 to include the latest publications. Databases searched included Library, Information Science & Technology Abstracts; Information Science & Technology Abstracts; Library & Information Science Source; and Education Source. The search strategy combined common phrases for library instruction (i.e., “library instruction,” “information literacy instruction,” and “bibliographic instruction”) with terms related to humor (i.e., “humor,” “comedy,” “jokes,” “funny,” and “laughter”). The search yielded both peer-reviewed and non-peer-reviewed publications, but the databases’ built-in limiters were used to exclude book reviews. To gain a better understanding of this topic’s prevalence in the literature over time, publication date was not used to limit the results. Cited reference searching and “pearl growing” were also used to identify publications that were not found through searching the databases. Publications that did not explicitly address the use of humor or that did not involve some form of library instruction were excluded.

In addition to searches of the library literature, the health sciences education literature was also searched using PubMed, MEDLINE, CINAHL, and Health Source: Nursing/Academic Edition in October 2018. As with the search of the library and information science literature, search terms related to humor were used alongside terms for education (i.e., “teaching” and “learning”). Database limiters were used to exclude book reviews and publications that focused solely on patient education.

## RESULTS

Humor has consistently appeared in the library and information science literature since the mid-1980s. This literature contains several common themes, including the benefits of humor in the classroom and librarians’ attitudes toward humor as well as librarians’ accounts of the most commonly used types of humor. Nurse educators authored most of the publications exploring humor in health sciences education. These articles highlight attitudes toward humor and several comedic teaching strategies that are comparable to those featured in the library and information science literature.

### Benefits of humor in the classroom

While most authors rely on anecdotal evidence in their discussions of humor’s benefits in the classroom, a handful of qualitative studies indicate that students appreciate funny teachers. Nevertheless, the same core arguments appear throughout the literature. Humor can make an instructor seem more relatable and approachable in the students’ eyes. It can also help create a positive, stress-free learning environment. Students are more likely to pay attention to a funny teacher, and some authors contend that the thoughtful use of humor strengthens students’ recall of the material and inspires creative thinking. Comedy may, moreover, help prevent or mitigate the effects of instructor burnout.

One of the most frequently mentioned benefits of integrating humor into library instruction is its ability to put students at ease and alleviate any stress or anxiety they may feel, which fosters a more open and relaxed classroom environment that is conducive to learning [1–3, 6–12]. Medical educators—especially those working in clinical settings—emphasize the anxiety-reducing power of humor [5, 13–25]. It can serve as an effective coping mechanism for students who are encountering such a high-stakes environment for the first time [14, 15, 25]. Health sciences students can also learn how to use humor to help their future patients manage stress [5, 13, 14, 22].

Comedy’s ability to keep students engaged is likewise an advantage. Warnken and Young note that the training and development literature acknowledges humor’s effectiveness at capturing an audience’s attention, and librarians can learn from this [26]. Many of the authors acknowledge that library instruction, while important, might not be an exciting topic, particularly in the eyes of college students. Student boredom can hinder learning, but humor has the power to assuage ennui and help direct students’ attention back to the instructor [10, 13, 24, 27]. This sentiment appears in the health sciences literature as well. Humor can help instructors liven up their lectures or maintain their students’ motivation during challenging courses or semesters [13, 16, 21].

In a similar vein, teachers can use humor to build rapport with their students. Such levity can make them seem friendly and approachable, which may mean students are more likely to ask their instructors for help when they need it [1, 3, 9, 11, 14, 23]. Several studies indicate that nursing students feel more comfortable around clinical instructors who regularly use humor, and, consequently, students are more willing to ask questions as well as admit to and learn from their mistakes [13–15, 18, 23]. Brewerton encourages the use of positive, empowering humor that humanizes the joke-maker or subject matter [10]. Likewise, Liebman states that comedy “humanize[s] and debureaucratize[s] the library” [28]. This goodwill may even translate into an increase in reference desk visits.

Several authors contend that humor increases students’ retention of instructional content [7, 8, 12, 14, 16, 20, 22, 23]. The empirical evidence for this—drawn primarily from the education literature—is mixed [4, 24, 25, 29]. Anecdotally, some instructors report that students remember content tied to a particular joke or humorous example when questioned at the end of a lesson [16, 21]. Students who are engaged and attentive are more likely to have absorbed the material to begin with, however. Some authors take this argument a step further and claim that humor inspires creativity and enhances problem-solving capabilities [1, 7, 10–12, 14, 20, 22, 30, 31]. Creative thinking can be especially useful in fast-paced clinical environments, where resourcefulness is paramount. It is, therefore, a skill that health sciences instructors are eager to develop in their students [14, 20, 22, 31]. Librarians can leverage this potential advantage to help students think through abstract, hard-to-grasp information literacy concepts [1, 11].

Another important benefit of using humor is its potential for keeping instructor burnout at bay [11, 3, 19, 30]. Teaching information literacy can, in some contexts, become fairly repetitive, and high-stakes clinical environments can wear down instructors as well as students [13, 19]. If teachers are bored, frustrated, or exhausted, their students are likely to sense this and become disengaged [30]. As is often the case with active learning, using humor and getting laughs can make instruction sessions more fun and pedagogically rewarding for instructors as well as students.

### Instructor attitudes toward humor

Three studies—conducted several years apart—examine librarians’ opinions of humor. In 1992, Osborne sent a questionnaire on humor and library instruction to 70 librarians working in the State University of New York system [32] and reported that respondents indicated an appreciation of humor’s benefits. They felt that it made them seem more approachable, put their students at ease, and helped them build rapport in the classroom [32]. Perret’s 2016 article likewise highlights the results of a survey of 55 librarians’ attitudes toward and use of humor. Librarians reported having very favorable views of humor and its benefits in the context of library instruction. Only 1 respondent thought that using humor in the classroom was inappropriate. Nearly all (96%) respondents reported using humor during library instruction sessions [3]. In addition, Marshall asked 21 librarians about their use of humorous examples from popular culture, such as comics and television sitcoms. The majority of those who incorporated funny references into their instruction sessions thought that it was successful at capturing students’ attention and creating a welcoming environment [9].

Health sciences instructors also value humor and report using it to some degree in their teaching. Ziegler polled senior staff at the Sydney Children’s Hospital and found that about 80% used humor during instruction [25]. More recently, Liu et al. surveyed 327 Chinese medical students and 165 physician teachers. Most (87%) respondents indicated that they supported the use of humor in the classroom [17]. The work of Nahas [18] and Ulloth [23] suggests that both nursing students and instructors appreciate and make use of humor.

Even though librarians hold an overwhelmingly positive view of classroom humor, many authors caution against the inappropriate use of comedy. Sarkodie-Mensah refers to humor that demeans individuals or particular groups of people (e.g., sexist, racist, homophobic jokes or jokes aimed at various ethnic groups) as “tasteless” humor and urges librarians to refrain from using this type of comedy [7]. The majority of the respondents in Perret’s study also viewed jokes targeted at individuals or groups as inappropriate. They listed “racy/bawdy humor” and “gross-out” among some of the other sorts of comedy that they thought librarians should avoid. Some respondents worried that students would perceive librarians as “out of touch” if their attempts at humor fell flat and thought that too much humor might prove distracting [3]. Sarkodie-Mensah [7] and Arnsan [8] likewise both warn against compromising on substance for the sake of getting another laugh. The students are there to learn, not to be entertained. These sentiments are echoed by others [1, 4, 10–12, 13, 30].

Several of the health sciences papers likewise caution against humor that targets individuals or specific groups of people. The authors also urge their colleagues to consider cultural differences when gauging the appropriateness of comedic material, perhaps more so than in the library literature [13–14, 17, 18, 21, 22, 24]. Nahas found that nursing students from non-Western cultural backgrounds were more likely than their peers to view clinical instructors’ humor use in a negative light [18]. Other authors worry about the use of “gallows humor” and the negative effect this can have on students [13, 33].

MacAdam is the only author to give voice to serious reservations about humor’s use in the library classroom. She cites research studies from the fields of education and communication that suggest that students perceive instructors who use planned humor (or seemingly planned humor) less favorably. This is compounded when the instructor is a woman, which should give practitioners in the female-dominated profession of librarianship pause. In spite of these reservations, she acknowledges that “there comes a time when a teacher faces students and common sense, instinct, experience, personal style, and professional conviction must temper research findings” [29].

### Cultivation of a sense of funny

Several articles stand out with regard to their approach to humor. Instead of touting the educational benefits of being funny during library instruction sessions or outlining specific examples of how to do so, Trefts and Blakeslee [34] consider how librarians themselves can become funnier—how they can “cultivate a sense of funny”—as do Fulton [1], Sarkodie-Mensah [7], Brewerton [10], and Vossler and Sheidlower [4]. Tewell examines the techniques that successful comedians use to engage their audiences and what instructor librarians can learn from them [35]. Health sciences educators suggest similar strategies for cultivating a sense of funny that are appropriate for their students. Taken together, these publications provide librarians with practical advice on developing a lighthearted yet assured classroom persona.

Trefts and Blakeslee enrolled in a four-hour stand-up comedy workshop at the Punch Line Comedy Club. They learned that comedians believed that having a sense of humor (finding something funny) and “having a sense of funny” (understanding what others will find funny) were different things [34]. The authors then used Booth-Butterfield and Booth-Butterfield’s Humor Orientation scale to rate how funny they themselves naturally were [36]. Despite realizing that they might not be as funny as they would like to be, Trefts and Blakeslee write that “you have to work with what you’ve got” and outline their advice for other librarians looking to bring more humor into their instruction sessions [34]. Vossler and Sheidlower echo this advice in their 2001 book *Humor and Information Literacy: Practical Techniques for Library Instruction*. They also suggest that librarians examine the techniques that professional comedians use [4]. Tewell likewise notes that librarians can learn a great deal from “comedians’ extensive experience in public speaking, engaging audiences, and performing for new faces night after night” [35]. Instruction librarians who are interested in developing their comedic potential can also benefit from spending time with and observing other funny individuals, especially teachers [7, 13, 17, 34].

Furthermore, instructors should get to know their audiences well and look for humor in their everyday lives that would appeal to these audiences. Understanding what a particular group of people—undergraduate nursing students, for example—will find funny is key and is something a liaison librarian might get a sense of, over time [7, 34]. Nahas found that nursing students’ cultural backgrounds affected how they felt about a clinical instructor’s use of humor. If possible, such characteristics need to be taken into account ahead of time [18]. Instruction librarians may find themselves speaking to an unfamiliar group, so knowing how to quickly read an audience is a worthwhile skill to develop [35]. Creating funny material can be a difficult undertaking, particularly for those just starting out. Thus, many of the authors recommend looking for and taking note of humor in everyday situations [1, 4, 7, 17, 34]. Several others suggest keeping a journal or file of anything that could be turned into a humorous joke, anecdote, or activity [1, 7, 13, 17, 34, 37].

Another key piece of advice is to be genuine. Humor should appear natural and spontaneous, even if it is carefully constructed and extensively practiced. Audiences will see through an overwrought performance or thinly veiled attempts at seeming “hip” [4, 34, 37]. The nursing students in Ulloth’s study were able to detect forced humor and felt that it negatively impacted their opinion of the teachers who tried it [23, 37]. Instructors should draw upon personal experience and chose styles of humor that reflect their personalities [1, 10, 11, 21, 22, 37]. Sarkodie-Mensah [7] and Vossler and Sheidlower [4] suggest developing a friendly and funny instructor persona but stress that this persona should closely align with a librarian’s underlying disposition. Librarians can exaggerate certain aspects of their personalities for comedic effect [4]. Sincerity helps instructors build rapport with students, which amplifies their “approachability factor” [35].

Finally, developing a working sense of funny necessitates practice and reflection. Librarians should start small, sprinkling a few jokes throughout their instruction sessions, but it is common for a joke to flop the first time. There is no need to abandon it after only one attempt: more time and effort may be required [34]. Reflection and feedback can help determine if a joke, anecdote, or activity is truly untenable; if it would be better suited to another audience or instructional context; or if it simply needs to be reworked [10, 34, 35, 37]. If so, more time devoted to planning and practice is key [4, 37].

### Examples from the literature

A major focus of the literature involves sharing specific examples. The examples fall under several broad categories: silly examples, false drops (searches that retrieve unrelated and unwanted results) and humorous analogies; costumes and props; storytelling; media and popular culture examples; jokes; and physical comedy. Of these, storytelling or role-playing, often enhanced by the use of costumes or props and humorous media (particularly cartoons), feature prominently in the health sciences.

#### Silly examples, false drops, and humorous analogies

By far the most popular form of humor in library instruction is the use of silly examples during in-class demonstrations of catalog or database searches [1, 2, 4, 8, 38, 39]. Vossler and Sheidlower devote a whole chapter of their book to searching with humor [4]. This technique only requires a little more time and effort on the part of the instruction librarian but can make the session more memorable for students [2]. Petry is a big proponent of this strategy for library catalog instruction and provides several examples in her article. She advocates the use of “whimsical catalog entries” or showcasing library materials that fit a particular theme. Examples can be mildly amusing or witty rather than laugh-out-loud funny [30]. Nurses Beitz [13] and Ulloth [37] also encourage word play and suggest instructors keep an eye out for puns, incongruities, and double meanings. Examples applicable to the health sciences include titles such as Oliver Sack’s famous *The Man Who Mistook His Wife for a Hat and Other Clinical Tales* and a quick joke from Brewerton regarding the “explode” feature in PubMed and MEDLINE: “Let’s look at exploding leg ulcers!” [4]. Meer et al. suggest showcasing obviously “bogus” websites during instruction sessions to demonstrate the importance of evaluation and critical thinking [39], an idea echoed by Ziegler in his article on humor in medical teaching [24]. Medical librarians can do something similar with sites that promote outrageous health information.

Librarians can also make use of false drops and humorous analogies in their teaching. False drops are humorous because they are unanticipated and catch students off-guard. They can also illustrate the importance of being intentional about search term and parameter selection. Whyte describes an instruction session during which she demonstrated a search for newspaper articles on the Cherokee people. The students were surprised to find that all of the results had to do with the Jeep Cherokee [38]. Humorous analogies also serve as a powerful teaching tool by likening one idea or image to another unrelated one [37]. Analogies, in and of themselves, are only “a tiny step away from humor” [4]. Librarians can use analogies to help explain abstract or difficult-to-grasp information literacy concepts, including those related to aspects of information creation and source evaluation [4].

#### Costumes and props

While not the most popular form of instructional humor in the literature, the use of costumes and props provides some of the most memorable examples. They can be built around a theme or subject area as in Smith’s “Perspectives on...the Pirate-Teacher.” Smith chose a pirate theme for a chemical information research skills course at Notre Dame University, likening searching databases to “searching for buried treasure.” She used the same theme for a first-year studies class, shifting the focus to “pirated” materials and copyright basics and academic integrity. Smith donned a pirate costume for both courses [27]. Antonelli et al. mention costumes in their article on using theatrical techniques to engage students during library instruction sessions. “Put on a Sherlock Holmes hat, and then add a magnifying glass and you can portray a library sleuth hot on the trail of a good research paper” is one example of costuming that they provide [40]. Given the right context, health sciences librarians can choose costumes that call to mind recognizable medical figures from history or pop culture.

Both Antonelli et al. [40] and Arnsan [8] suggest using props to catch students by surprise and quickly infuse a library instruction session with humor. “Punny” props that somehow relate to and will later remind students of a topic, concept, or tool discussed during the session are ideal. Librarians should be thoughtful about their use of props, however. Too many or too elaborate props can be distracting. They are tools and should relate directly to the subject matter [4]. Nevertheless, both Antonelli et al. [40] and Arnsan [8] reference rubber chickens, a classic comedy prop with no obvious information literacy tie-in. Arnsan takes his use of props a bit further. For example, he presented his classes with the *U.S. Budget in Brief* wrapped in a pair of men’s underwear and a fish cut out of old microfiche. However, Arnsan notes that he did this in a semester-long information literacy course and only brought out some of his more peculiar props once he established a good rapport with the students [8].

Only a few instances of costuming and the use of props appear in the health sciences literature. However, they often serve as key components of skits and role-playing activities [16, 21]. Others use props to capture their students’ attention. Beitz wore a Viking helmet to set the stage for a lesson on perioperative management [13]. Chiarello donned a clown nose for his first meeting with the nursing students in his clinical experience group to tame their stress levels and gauge their reactions to his sense of humor [14]. Wylie, on the other hand, provides a less ostentatious example of costuming: Faculty teaching a trauma nursing course coordinated their outfits around holidays or themes—St. Patrick’s Day, for example—to lighten the mood and put students at ease [41].

#### Storytelling and role-playing

Basing instruction on an amusing narrative, problem, or scenario is another option. Christian, for example, explores the many virtues of using stories to engage law students and foster the development of critical thinking skills in her 2014 article, “The Art of Storytelling.” Arguing that “[p]eople remember stories because they involve visualizing those involved and what happened to them,” Christian describes a semester-long class on advanced legal research that she taught and how she developed assignments around scenarios that she wrote. Some of these stories featured zany characters or situations. Her students then had to respond to these stories as if they were real cases that they had to research and work through [42]. Some of the other librarian authors also recommend the use of funny—or at least quirky—stories or anecdotes [6, 43].

Storytelling, and the related techniques of role-playing and skits, also appears throughout the nursing literature. Some authors contend that weaving humor into personal anecdotes, interesting cases, and role-playing exercises engages students and reinforces the concepts being taught, provided that they relate to the course material. Liu et al. found that students rated “interesting clinical cases” as the most effective form of humor [17]. Stories can help students develop empathy for patients. Parrott describes how she role-played a wise-cracking nursing home resident to highlight medical issues that are unique to the elderly [20].

However, teachers must avoid humor that hinges on hurtful stereotypes. Overcash advises instructors to tell stories that feature themselves as the main character of a story or role-playing activity for this reason [44]. Lee and Lamp, for instance, took turns visiting class dressed up as expectant mothers in need of prenatal and postpartum care information. One pretended to be a rabid hockey fan and donned a Toronto Maple Leafs jersey to capture the students’ interest [16]. Skits are another alternative. Smith and Noviello used faculty skits to illustrate key evidence-based practice concepts. One of these skits involved a group of faculty play-acting a road trip and connected the characters’ evaluation of various sources of information for directions to a discussion of library search techniques [21].

#### Media and pop culture

Several authors describe using humorous media during library instruction sessions or weaving funny pop culture examples into their teaching [1, 13, 22, 23, 34, 37, 43]. Marshall argues that pop culture examples can help make instructional content resonate with students. Currency, however, is key. Students will not laugh at references that they do not recognize. Most of the librarians who responded to Marshall’s survey used some type of pop culture material, with comics or cartoons, feature films, and television as the top three media formats [9].

Music and video clips can serve as effective icebreakers at the start of an instruction session or to introduce an important idea or concept. Meer et al. [39] and Trefts and Blakeslee [34], among others, suggest using music as an opener. The music can be humorous through its lyrics or by virtue of its tone or genre. Instructors can also compose their own music. A nursing faculty member featured in Ulloth’s study wrote rap songs to help her students remember key concepts [23].

Likewise, funny videos can be used as teaching tools. Tewell successfully used clips from television comedies to teach complex information literacy concepts to first-year students [45]. Liebman describes a series of “silent comedy” films created by the California State University, Los Angeles, library in the early 1980s. The movies’ protagonist was a “Chaplinesque” run-of-the-mill library patron who encountered obstacles commonly experienced when seeking information. The films introduced students to the library, while putting them at ease and showing them the human side of the institution [28].

Cartoons are another favorite teaching tool of both health sciences instructors and librarians. Several authors cite cartoons as a quick way to work comedy into a lesson [13, 17, 20, 22, 23, 26, 27, 41, 46]. Smith, for instance, uses cartoons as library instruction sessions openers and on assignment sheets and handouts [27]. Pease describes classroom activities built around cartoons, using them to communicate new nursing concepts and to help students scrutinize common stereotypes of nurses and their patients. One activity involved students writing their own captions for cartoons, which fostered critical thinking and facilitated discussion [46]. Beitz used cartoons and photographs to introduce serious issues in surgical nursing, such as the uncertainty and fast-paced nature of the work. The funny opening helped encourage discussion of difficult topics [13].

#### Jokes

Jokes—what most think of as humor—are riskier. Instructors must be familiar with their audiences and have a solid grasp of their sense of humor. Misunderstood jokes do not land well [10]. “A failed joke is quite a bit worse than no joke at all,” warn Vossler and Sheidlower [4]. While practice helps with delivery, maintaining the appearance of spontaneity is wise [11]. Jokes that seemed rehearsed are likely to fall flat [23]. As noted above, nearly every author advised against telling jokes that put down groups or individuals. However, negative humor that disparages something (not someone!) that is feared can help relieve tension [10]. Many teachers tell jokes as icebreakers or tie them to a larger theme [4, 13, 20, 41]. Smith, for instance, crafted silly, lighthearted jokes and riddles to fit the pirate theme of her classes [27]. Walker [11] and Ulloth [37] both suggest that instructors have quips ready for technological malfunctions and other instructional mishaps. Arnsan told jokes to highlight legal resources in his research skills course [8]. Vossler and Sheidlower devote a whole chapter of their book to different jokes [4].

#### Physical comedy

Very few instructors report incorporating physical comedy into their teaching. This type of humor is meant to grab students’ attention by surprising them. Vossler and Sheidlower describe miming, performing somersaults, and purposefully dancing badly during instruction [4]. Arnsan’s props involve some light physical comedy, in the form of an “exploding can of snakes” and a thrown rubber chicken [8]. One of the nursing instructors profiled in Ulloth’s 2002 article performs something called the “renal cheer” for her students but does not describe it in detail [23]. Southam and Schwartz discuss “kinesthetic humor”—humor that involves movement—in occupational therapy education and provide a few examples, such as adding a silly twist to an exercise to develop hand skills. They also include skits and role-playing in this category [22]. Physical comedy requires athleticism on the part of the instructor as well as adequate classroom space [4]. While these techniques can successfully redirect students’ attention, they are seldom related to the content of the session. Thus, physical comedy is the least written-about form of instructional humor.

## DISCUSSION

What does the literature tell us about humor and health sciences library instruction? Incorporating humor into instruction is an inherently risky endeavor, but the evidence indicates that—undertaken with care—it is a worthwhile one, especially given the high-stress nature of health care. Health sciences librarians will reap comedy’s reported benefits when choosing lower-stake forms of humor that directly relate to the content of the instruction session. They can draw upon strategies to foster a positive classroom environment, build rapport with students, and encourage creative thinking without hindering student learning.

Knowing one’s audience is key, since humor is contextual. Librarians who serve as liaisons are well equipped to evaluate the appropriateness of potential strategies for the disciplines they serve. Even so, it is best to start small. Librarians can begin by cultivating a warm, lighthearted instructor persona. They can take note of anything funny they encounter in their day-to-day work that they think a particular group might respond to and practice deploying it with a supportive colleague. Health sciences librarians who are interested in honing their humor skills should also seek out, observe, and collaborate with health sciences instructors who successfully use comedy in the classroom. They can work together to compile humorous cartoons or case studies and create compelling stories, skits, or role-playing activities to help their students learn important concepts and skills.

The most relevant—and relatively “safe”—comedic forms that health sciences librarians can begin integrating into their instruction sessions are silly examples, false drops, and funny media. Although they do not feature much in the health sciences literature, silly examples and false drops are easy to seek out and weave into an instruction session without wasting too much time or straying too far from the material.

Cartoons, a favorite tool of both librarians and their colleagues in the health sciences, are also worth trying. Librarians can open instruction sessions with a funny cartoon that illustrates an information literacy concept in a humorous way, which will likewise capture students’ attention without losing much teaching time. Alternatively, they can develop activities around cartoons, such as having students write captions for cartoons related to research, source evaluation, or critical appraisal.

Bolder librarians can turn to storytelling, role-playing, and skits. For example, they can have students role-play practitioners and patients as they learn to dissect a clinical question and search PubMed or dress up as a patient themselves. Developing and acting out humorous skits—perhaps alongside course instructors—is another option. Librarians’ examples need not be laugh-out-loud funny. Instead, they can create case studies with quirky characters in imaginative or entertaining situations, with whom the students can relate, and then base their instruction sessions on these examples. Although storytelling strategies require more time and planning upfront, they can add a spark to instruction sessions featuring problem-based learning or team-based learning scenarios.

Health sciences librarians who wish to keep their audiences engaged during instruction sessions, create an inviting classroom environment, and build rapport with students in their areas should consider adapting some of the humorous strategies described in the literature. Many librarians and health sciences instructors integrate such strategies—from cartoons and intriguing case studies to role-playing and skits—into their teaching to great effect. Their students likewise appreciate the levity that they bring to learning situations that can be quite stressful at times. While cultivating a “sense of funny” and successfully deploying humor can be challenging and require a good deal of practice and patience, the common threads in the library and information science literature and health sciences literature provide a roadmap for librarians.
